# The Nocebo Effect

**DOI:** 10.1146/annurev-pharmtox-022723-112425

**Published:** 2023-08-16

**Authors:** Luana Colloca

**Affiliations:** Department of Pain and Translational Symptom Science and Placebo Beyond Opinions Center, School of Nursing, University of Maryland, Baltimore, Maryland, USA;

**Keywords:** negative outcomes, expectancy, discontinuation, framing effects, nonadherence, learning, side effects

## Abstract

Adverse nocebo responses can cause harm to patients and interfere with treatment adherence and effects in both clinic practice and clinical trials. Nocebo responses refer to negative outcomes to active medical treatments in clinical trials or practice that cannot be explained by the treatment’s pharmacologic effects. Negative expectancies and nocebo effects are less known than placebo responses. Nocebo effects can be triggered by verbal suggestions, prior negative experiences, observation of others experiencing negative outcomes, and other contextual and environmental factors. As research advances over the years, mechanistic knowledge is accumulating on the neurobiological mechanisms of nocebo effects. This review summarizes studies on different facets of nocebo effects and responses and discusses clinical implications, ethical considerations, and future directions.

One wonders how often a useful drug has been discarded because of “toxic effect” in the first trials which have been due to the accident of there being an appreciable number of nocebo reactors in the test subjects.

—W.P. Kennedy, 1961 (1, p. 204)

## 1. INTRODUCTION

Patients receiving placebos in randomized clinical trials often report side effects that mirror those reported by patients receiving active medical treatment. These effects are called nocebo responses and are attributed to the communication of potential adverse effects during informed consent (2). The term nocebo was introduced by Kennedy (1) in 1961 to specifically denote the nocebo reaction as the negative counterpart of the positive placebo reaction or response (1, 3).

Nocebo responses are adverse responses to placebo interventions in placebo-controlled trials. Nocebo effects are neurobiological phenomena associated with actual or perceived harm. These negative effects can occur because of negative expectancies due to verbal suggestions, prior learning-based experiences, social observation, mass psychogenic modeling, negatively perceived patient-clinician communications, and clinical encounters (4–7). Nocebo responders are participants who respond negatively to a placebo treatment or intervention. Thus, the concept of nocebo effects has recently drawn attention in both basic and clinical research (1, 8).

Here, results from mechanistic research on nocebo effects and responses are reviewed, and implications for clinical practice and ethical considerations are discussed. In addition, how verbal communication, environmental factors, and prior experience with unsuccessful treatments can induce negative expectancies that cause nocebo effects is also discussed. Nocebo effects are generated by biological mechanisms (9), and behavioral, brain imaging, and pharmacological studies have helped to identify mechanisms responsible for these effects. The neurobiological mechanisms of nocebo effects are not yet fully understood, but several theories attempt to explain how they work. One widely accepted theory suggests that the nocebo effect is mediated by similar neurobiological pathways as placebo effects. Nocebo effects are the result of the brain’s response to expectancy and anticipation of negative outcomes, which triggers the release of stress hormones and other neurotransmitters, leading to a range of physical and psychological symptoms. Additionally, social and cultural factors can also influence nocebo effects.

## 2. NOCEBO RESPONSES IN RANDOMIZED CLINICAL TRIALS

In early research, nocebo responses were regarded by some as an inconvenient phenomenon that made it harder to validate medications because of the occurrence of side effects in the placebo arms. In randomized clinical trials, nocebo responses can decrease treatment compliance, cause treatment discontinuation, and increase the need for higher doses and/or sample sizes to show treatment efficacy (6).

Treatment discontinuation is a common phenomenon in both randomized clinical trials and clinical practice and is often associated with patient-reported or actual adverse events (10). Myers et al. (11) reported that communicating to angina pectoris patients about the potential gastrointestinal side effects of aspirin and sulfinpyrazone can lead to withdrawal from the study. Cairns et al. (12) conducted secondary retrospective data analyses to quantify the effect of merely communicating gastrointestinal side effects in the consent form. The effect of disclosure of gastrointestinal side effects on patient adherence and observed side effects was assessed in a randomized, double-blind, placebo-controlled trial that tested the benefit of aspirin, sulfinpyrazone, or both drugs for unstable angina pectoris (11). The patients who were informed in the consent forms about the potential gastrointestinal side effects experienced in a sixfold larger increase in gastrointestinal symptoms and patient-initiated discontinuation of treatments (11).

Retrospective secondary analyses of nocebo responses (and dropout rates) have been conducted for randomized controlled trials of multiple disorders, including multiple sclerosis (13, 14), neuropathic pain (15), fibromyalgia (15), motor neuron diseases (16), depression (17), migraine (18), headache (19–21), tension-type headache (19), and osteoporosis (22).

Treatments for migraine (23), depression (24), and high cholesterol [e.g., statins (10)] have been studied for their high rates of side effects observed in the placebo groups of randomized clinical trials. Systematic reviews of randomized, double-blind, placebo-controlled studies for migraine treatments indicate that side effects associated with placebo interventions often match those associated with the treatment drugs (23, 25). Amanzio et al. (23) conducted a systematic review of side effects in the placebo groups of 69 trials of antimigraine treatment, including 56 trials for triptans, 9 trials for anticonvulsants, and 8 trials for nonsteroidal anti-inflammatory drugs. High rates of side effects in the placebo groups of the trials matched those described for the treatment groups. Anticonvulsant placebos produced anorexia, memory difficulties, paresthesia, and upper respiratory tract infection—all side effects typical of antimigraine treatments (23). The link between the reported side effects in the placebo groups and the known side effects of the treatments suggest a genuine nocebo response that is related to disclosures in the informed consent.

Rief et al. (24) compared side effects in the placebo groups of two classes of antidepressants: tricyclic antidepressants (TCAs) and selective serotonin reuptake inhibitors (SSRIs). This systematic review and meta-analysis included 143 placebo-controlled randomized clinical trials with 12,742 patients. Higher rates of side effects were reported in placebo-group subjects in TCA versus SSRI trials, including dry mouth, drowsiness, constipation, and sexual problems. Higher rates of side effects were detected by systematic versus less systematic assessment of side effects. These side effects were influenced by patients’ and investigators’ expectations, again suggesting a link between informed consent and side effects (24).

A recent lipid-lowering component of the Anglo-Scandinavian Cardiac Outcomes Trial explored the side effects of atorvastatin (26). The study recruited 10,180 patients with hypertension, three cardiovascular risk factors, and 5–6 mmol/L or lower fasting total cholesterol concentrations. The participants were taking a statin or fibrate, had no clinical history of myocardial infarction, and were not treated for angina (26). They were assigned to either atorvastatin 10 mg daily or placebo in a randomized, double-blind, placebo-controlled Phase III trial. In the subsequent nonrandomized, nonblinded Phase IV extension, all patients were offered open-label atorvastatin 10 mg daily. In a blind fashion, four side effects (muscle-related, sleep disturbance, erectile dysfunction, and cognitive impairment) were analyzed. Both the 10-mg open-label atorvastatin and placebo resulted in an increased rate of muscle-related side effects, but only in the three-year nonblinded, nonrandomized follow-up phase (26). In the initial five years of the randomized, double-blind, placebo-controlled trial, the participants showed no increase in side effects. Thus, the authors proposed that the excess rate of muscle-related side effects (a well-known side effect of statins) occurred only when patients and their physicians knew that atorvastatin was being administered.

Public perceptions of medication side effects may also contribute to nocebo reactions to antidepressants (27, 28) and thyroxine, used to treat hypothyroidism (29). In 2007 and 2008, pharmacies in New Zealand switched to a new formulation of thyroxine branded as Eltroxin. Since 1973, Eltroxin, made by GlaxoSmithKline, had been the only thyroid hormone approved and funded by the New Zealand government. In 2007, the company moved the manufacture of Eltroxin from Canada to Germany. The active ingredient (thyroxine) continued to be made in Austria with no changes. However, the excipient (i.e., a tablet’s inert ingredients) changed in terms of color, size, markings, taste, and tongue dissolution rate. Once the new tablets were introduced, the side effects rate rose nearly 2,000-fold from 14 reports in 30 years to more than 1,400 in 18 months (29). Similar mass psychogenic effects have been reported for wind turbines and headache experience in the New Zealand population (30, 31).

In summary, nocebo responses, which are negative responses to inactive treatments or intervention with nocebo information, can decrease treatment compliance, increase sample sizes needed to show treatment efficacy, and cause treatment discontinuation. These responses have been found in randomized clinical trials of various diseases, such as multiple sclerosis, neuropathic pain, fibromyalgia, depression, and migraine. Informed consent disclosures in consent forms appear to be linked to nocebo responses. The reported side effects in placebo groups often match the known side effects of the treatments, suggesting a genuine nocebo response. Public perceptions of medication side effects may also contribute to nocebo reactions (4). As discussed below, nocebo responses can also influence decisions to use biosimilar products (32).

A new avenue of nocebo research has opened with the introduction of biosimilar products (33). Biosimilars are biologic medical products containing the same active ingredient as the originator biologics (originator products) but are manufactured by a different company. Currently, in rheumatology and oncology, nocebo responses may prevent patients from switching from originator biologics to biosimilars (8, 34).

In the DANBIO registry, rheumatologists collect data as routine practice. The disease status was similar before and after patients with rheumatic disease switched from the originator infliximab to its biosimilar CT-P13 (35). However, the adjusted retention rate after one year was significantly lower than the retention rate of those receiving the originator infliximab (36). These findings suggest a possible nocebo response.

Similarly, Boone et al. (35) investigated negative therapeutic outcomes following the switch from the originator infliximab to the biosimilar CT-P13, as well as the effects of reinitiating the originator infliximab in chronic immune-mediated inflammatory diseases such as irritable bowel syndrome (IBS) and rheumatic diseases. The effectiveness, immunogenicity, and tolerability profiles of the originator and biosimilar infliximab were comparable, but negative outcomes were observed in 13% of patients who switched from the originator to the biosimilar (35). Both IBS and rheumatology patients self-reported infusion reactions and headache, suggesting that nonmedical switching may have caused nocebo responses.

In two observational prospective studies of patients with rheumatic disease consenting to switch from the originator infliximab to the biosimilar CT-P13, improvements in disease status were comparable between therapies one year after the transition (8, 37). However, in one study (37), 15% of patients discontinued CT-P13 treatment despite having no worsening of disease. In the other study (38), 25% of patients discontinued the biosimilar due to subjective worsening of disease status and/or tolerability. Similarly, a recent systematic review that compared outcomes when switching from the originator biologic to biosimilar products in open-label and double-blind fashion indicated higher discontinuation rates, adverse events, and lack of efficacy in infliximab biosimilar open-label studies versus double-blind studies (39).

The studies suggest that treatment discontinuation upon a change in formulation is more complex than that seen in a randomized, controlled trial. The indication for discontinuation in these studies varies, and it may be due to subjective worsening of disease status and/or tolerability issues rather than objective worsening of the disease. Therefore, it is important to consider these factors when evaluating the efficacy and safety of biosimilars and other medications.

Professional societies such as the European Society for Medical Oncology (40) and the American Society of Clinical Oncology (41) have emphasized the importance of education and patient-clinician dialogue for the acceptance of biosimilars. In a guide for nurses involved in switching patients between similar biological medicines, the European Specialist Nurses Organisation specifically address the problem of nocebo responses, providing information on how to respond to patients’ questions about biosimilar cost/quality and potential nocebo responses and how to advise patients about factors such as trust and beliefs about good efficacy (42).

Overall, these studies suggest that treatment discontinuation upon a change in formulation is more complex than that seen in a randomized controlled trial and may be due to subjective worsening of disease status and/or tolerability issues rather than objective worsening of the disease. Educational programs can potentially help minimize nocebo responses (33), with an emphasis on the mode and content of communication strategies (43). Research on message framing and patients’ perception can advance educational programs in tandem with the development of patient-centered new policies (44–46).

## 3. MECHANISMS OF NOCEBO EFFECTS

**Figure 1:**
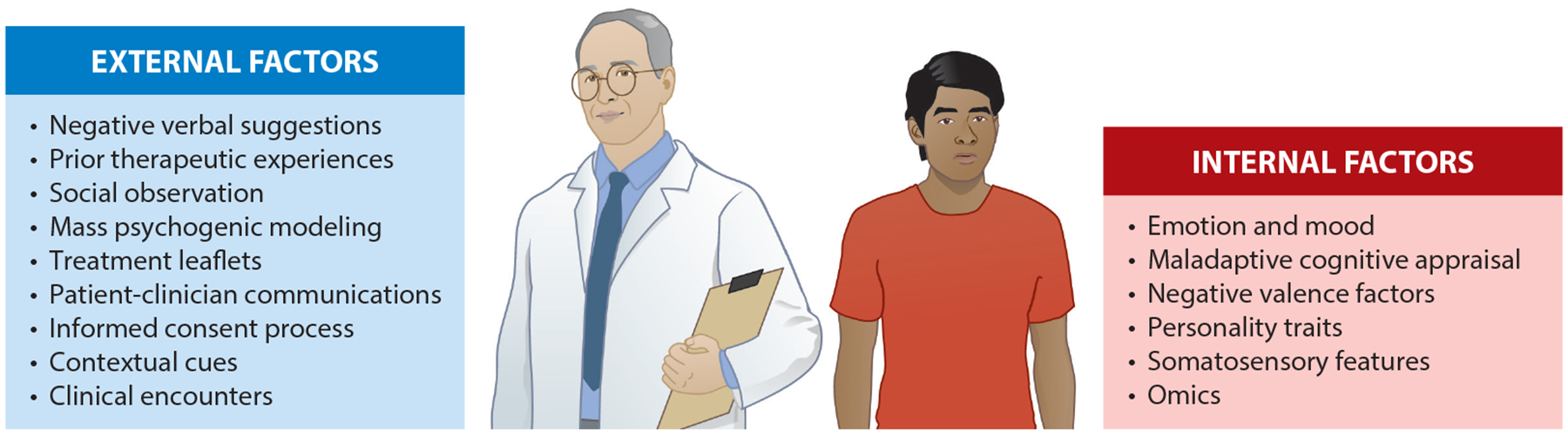
Schematic representation of potential external and internal factors triggering nocebo reactions. Verbal suggestions (e.g., the treatment has been stopped), prior experiences (e.g., exposure to increased painful intensities), social observation (e.g., seeing someone suffering from a side effect), mass psychogenic modeling (e.g., believing wind turbines induce headache), treatment leaflets (e.g., list of side effects), patient-clinician communication (e.g., “this procedure is going to be painful”), contextual cues (e.g., smelling a chemotherapy), and overall clinical encounters are examples of external factors that trigger nocebo reactions. In contrast, negative mood and emotions, negative valence factors, maladaptive cognitive appraisal, personality traits, somatosensory features, and omics are among the internal factors that can share nocebo responsivity. Figure adapted from Reference 47.

The observation that participants can experience side effects after being given placebo tablets, pills, or injections or undergoing sham procedures and surgeries raised interest in understanding the mechanisms of nocebo effects. Internal and external factors contribute to nocebo effects. Negative expectations can be linked to negative verbal suggestions (e.g., the treatment has been stopped), prior experiences (e.g., exposure to increased intensity of pain), and social observation (e.g., seeing someone suffering from a side effect). Mass psychogenic modeling (e.g., belief that wind turbines induce headache), treatment leaflets (e.g., lists of side effects), patient-clinician communication (e.g., “this procedure is going to be painful”), and clinical encounters can also trigger nocebo effects. Internal factors include individual patients’ expectations, genetic make-up, cognitive appraisals, and negative mood and emotions (Figure 1).

Mechanisms responsible for nocebo effects were investigated in clinical and experimental settings with some ethical restraints due to negative and potential harmful conditions (6, 48). In clinical trials and experimental human research of nocebo effects, nocebo responders are those who respond negatively to positive suggestions and/or placebos given to elicit an improved outcome. Nocebo responders are also those who respond negatively to verbal suggestions, negative modeling, and learning-based procedures designed to create anticipatory expectancies.

For both placebo and nocebo effects, the proportion and characteristics of placebo/nocebo responders remain unknown (49–54). These questions are also applicable to the nocebo effect. Regarding characteristics associated with nocebo effects, a recent study involving 624 healthy participants undergoing a placebo and nocebo (heat pain) procedure reported that nocebo responses were associated with neuroticism and the thermal pain threshold (54).

**Figure 2:**
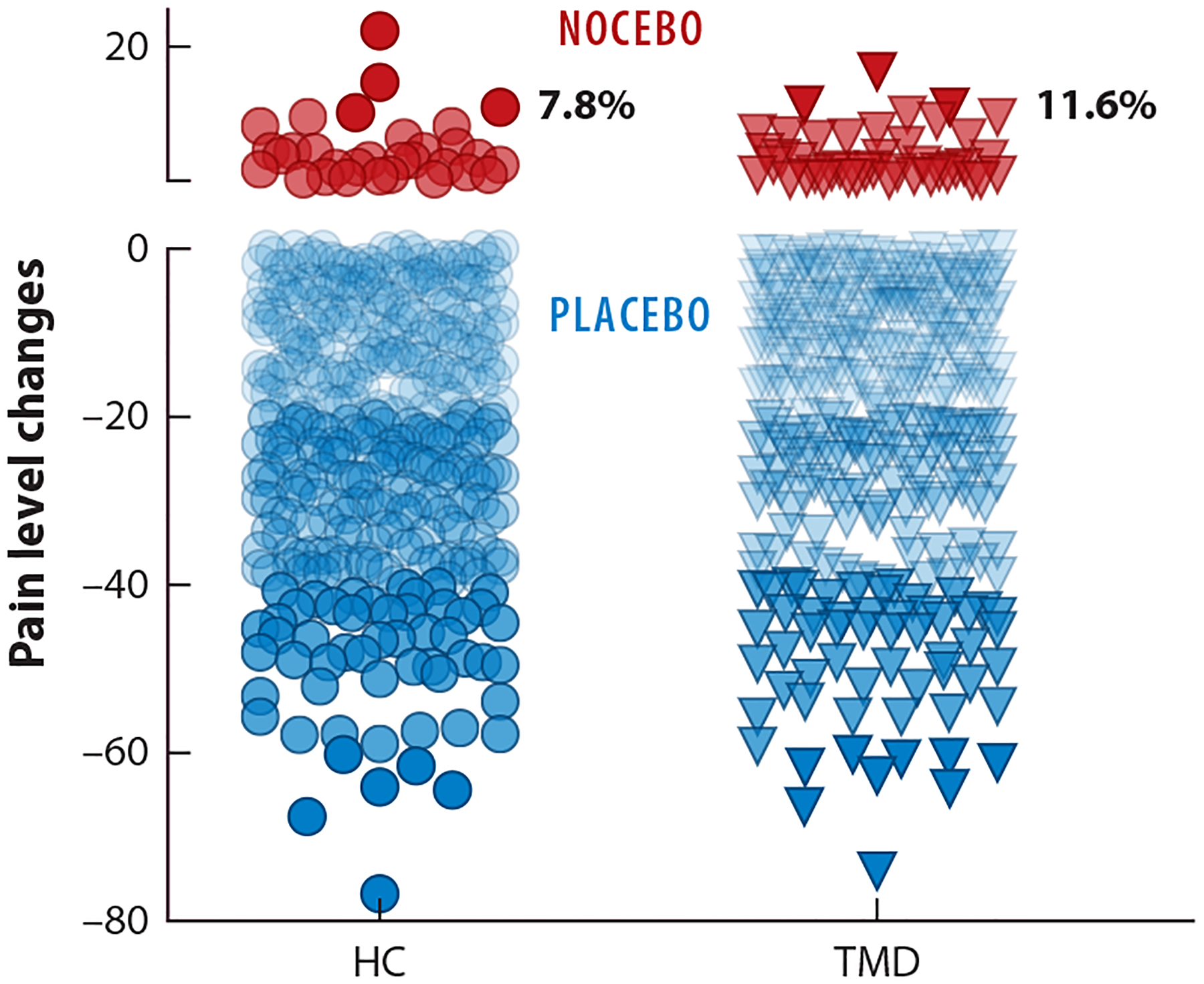
Individual differences in nocebo effects. The figure shows the distribution of placebo (*blue*) and nocebo (*red*) response in participants who underwent a placebo procedure. Study participants included healthy controls (HC) and chronic pain participants suffering from orofacial pain due to temporomandibular disorders (TMD). The placebo manipulation included verbal suggestions of pain reduction and conditioning in which the same painful thermal stimulus was paired with distinct visual cues to assess placebo effects. A sham electrode was attached to the forearm to make participants believe that their pain sensation would decrease. Differences in pain report were operationally defined as placebo (reduction) or nocebo (increase) responses. Each point represents the change in pain levels expressed as the difference score between control and test trials in 400 healthy (*circles*) and 363 chronic pain (*triangles*) participants. The percentage of responders for each range of pain level changes is shown to the right of the individual data. Each point (*circles* and *triangles*) represents the average delta score between six control and six placebo trials. Data from Reference 55.

Every study has some participants who respond to placebos in a negative way. We assessed the proportion of nocebo responders in a cross-sectional study with healthy, pain-free and chronic pain participants. Nocebo responders are those who responded negatively to a positive placebo manipulation consisting of verbal suggestions and conditioning (55). We found that 7.8% and 11.6% of participants, respectively, reported high pain following the placebo manipulation (Figure 2). Nocebo responders were characterized by a higher level of fear of pain, catastrophizing, and emotional distress (55). This finding is consistent with the proportion of treatment discontinuations (dropouts) observed in the clinical trials discussed above.

In terms of demographic characteristics, sex has been reported as a predictor of nocebo. Existing evidence on sex differences in nocebo effects is often inconsistent (56, 57). Additionally, several important factors have not been considered in previous systematic reviews and meta-analyses on this topic (i.e., there is a lack of studies that report sex differences). One review that combined studies with healthy participants and those with IBS and social phobia found that men tended to experience more placebo effects, while women experienced more nocebo effects (58). The type of manipulation used to induce placebo effects in the lab seemed to be relevant in observing sex differences, with men showing greater effects from verbal suggestions and women showing greater effects from conditioning (59). Observations like these have driven the recent interest in defining mechanisms of nocebo effects and potential risk factors associated with these effects, as described below.

### 3.1. Behavioral Mechanisms of Nocebo Effects

Behaviorally, classical conditioning, partial reinforcement, and observational and verbally based learning (i.e., suggestions) were tested to determine psychological mechanisms of nocebo effects (60).

**Figure 3:**
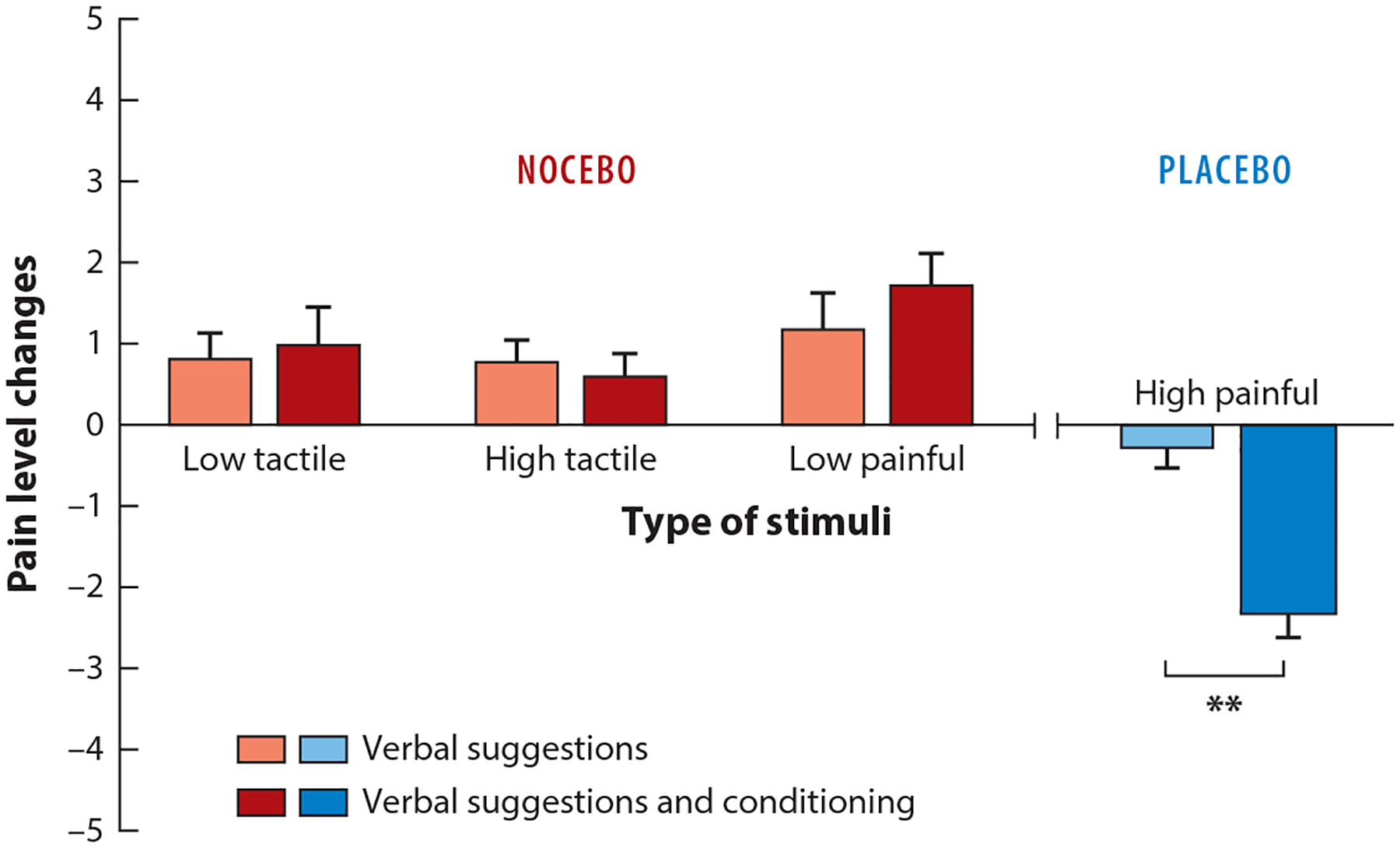
Nocebo allodynia and hyperalgesia. The bars show the mean response and standard deviation in participants who received verbal suggestions alone versus participants who underwent a conditioning procedure in the three nocebo conditions (e.g., type of stimuli). The three nocebo conditions were low tactile, high tactile, and low painful stimuli. Verbal suggestions alone and conditioning elicited the same magnitude of nocebo responses across the three conditions. Tactile stimuli were perceived as painful (allodynia) and low painful stimuli were perceived as high painful stimuli (hyperalgesia) as a result of nocebo effects. In contrast, in the placebo condition, verbal suggestions and conditioning showed significant differences (** indicates that *p* < 0.01). Data are expressed as mean average and error standards. Data from Reference 63.

Nocebo-induced hyperalgesia—a higher subjective pain perception after a patient or study participant receives a placebo (e.g., a fake cream), undergoes a sham surgery (e.g., a cut on the skin), or hears even the mere mention of pain increase—has been used as a model to study nocebo mechanisms (61). Colloca et al. (62) compared the effects of verbal suggestions alone (i.e., no prior pain experience) and classical conditioning (i.e., preexposure) to high painful stimulations. Negative suggestions regarding an upcoming high painful stimulation were used along with either just-detectable or low-intensity painful electrical shocks. Results showed that participants who were merely informed about the upcoming high pain but received just-detectable stimulations perceived the stimulations as more painful than those who were preexposed to high painful stimulations (Figure 3). When painful stimulations were used, both verbal suggestions alone and verbal suggestions with conditioning induced similar magnitudes of nocebo hyperalgesia (62). The finding that nocebo effects are of comparable magnitude when verbal suggestions and conditioning are used is different from what has been found for placebo effects, where conditioning has stronger effects than verbal suggestions (63). These results were replicated in subsequent studies (64, 65).

Regarding conditioning, one session of conditioning is sufficient to induce nocebo effects that are similar in magnitude to those of four sessions (66). Therefore, it is not necessary to have prior experiences of hyperalgesia to have nocebo hyperalgesia (66). It has also been shown that nocebo effects can be elicited by conditioning alone, that is, without any verbal suggestions (67).

Previous studies have also investigated the effects of partial and continuous reinforcement on nocebo hyperalgesia (68). Partial reinforcement refers to a schedule in which some, but not all, trials are reinforced by the unconditioned stimulus. Behaviors acquired under partial reinforcement tend to extinguish more slowly than do behaviors learned under continuous reinforcement, a phenomenon called the partial reinforcement extinction effect (69). In the case of nocebo hyperalgesia, partial reinforcement elicits smaller responses compared to continuous reinforcement, but these responses are more resistant to extinction (68). Moreover, preexposure to pain has been found to reduce nocebo effects (70).

Social observation (e.g., watching a video of or seeing a confederate, someone else, or a group experiencing negative outcomes) can also induce robust nocebo effects (71–78). In contrast, operant learning did not result in nocebo responses when an operant avoidance learning task was used (79).

An important question is whether nocebo effects generalize within (e.g., from heat- to pressure-induced nociception) and across (e.g., from pain to itch) modalities. Nocebo hyperalgesia induced by verbal suggestions generalizes from one form (e.g., heat pain) to another form (e.g., pressure pain) of pain but not from one modality (e.g., pain) to another (e.g., itch) (80).

Overall, humans can feel nocebo-induced hyperalgesia (81) as a result of simply being told about potential painful stimulations as a result of conditioned responses (e.g., associations of the colored light with high painful stimulations) and/or of labeling characteristics (e.g., prize, labeling, and marketing) (82). From an evolutionary perspective, nocebo (aversive responses) and placebo (safety responses) pathways may represent two opposite learning mechanisms that coexist in an organism. Nocebo effects may induce short-term defensive responses that enhance perceptual processing and negative outcomes, while placebo effects are long-term responses that may favor positive outcomes.

### 3.2. Brain Imaging Studies of Nocebo Effects

Behavioral manipulations have helped to identify the learning mechanisms of nocebo effects. To parallel behaviors with neural changes in the brain, brain imaging studies have combined behavioral procedures with functional magnetic resonance imaging (fMRI) approaches to shed light on pathways associated with increased pain (and other symptoms), negative expectations, and nocebo-related outcomes.

For example, nocebo effects can depend on the order in which treatments are delivered (63, 83). Kessner et al. (83) showed that starting a new medication after a prior unsuccessful medication induces a nocebo effect (i.e., pain increases) and is associated with the activation of the posterior insular cortices, areas associated with nociception and pain increases.

Tinnermann et al. (84) showed that labeling an inert treatment as an expensive treatment leads to stronger nocebo hyperalgesic effects than labeling it as a cheap treatment. Nocebo effects are associated with connectivity changes among prefrontal areas, brainstem, and spinal cord. The results showed that the prefrontal cortex was more active when participants thought the treatment was expensive. Additionally, the rostral anterior cingulate cortex (rACC) and the periaqueductal gray (PAG) were more active when participants believed the more expensive treatment would have more pain-related side effects (negative expectations). This process may have increased nociception in early subcortical areas and the spinal cord, which is also involved in placebo-induced pain reduction. The rACC predicted the magnitude of nocebo hyperalgesic effects and showed deactivation, favoring subsequent activation of the PAG and spinal cord, resulting in increased nociceptive inputs. Therefore, the study suggests that the rACC-PAG-spinal axis may be responsible for the effects of pricing on nocebo hyperalgesia. These results extend those from a prior study showing a potential role of spinal cord mechanisms and descending modulatory systems in negative expectations and, subsequently, nocebo hyperalgesia (85). When people have negative expectations about a drug due to its features or their past experiences with unsuccessful treatment or because they have heard about adverse effects from others, it can lead to nocebo effects. These effects can influence human behavior and lead to negative clinical outcomes (81).

The dorsal horn of the spinal cord receives nociceptive inputs that are regulated by descending control regions such as the rACC, hypothalamus, amygdala, and PAG. The rostroventral medulla in the brainstem combines these areas to influence nociceptive processing, and the output is determined by the behavioral circumstances (86–89) and involves the endogenous opioid system (90, 91). This can either have an antinociceptive effect or facilitate nociception. Increased activity within the parahippocampal, entorhinal, and brainstem network due to heightened anxiety and anticipation can intensify the pain experience (92, 93). According to a pioneering neuroimaging study, the hippocampus is involved in nocebo effects (94). Namely, the hippocampus and regions associated with anticipatory anxiety are associated with nocebo effects (95). That is, nocebo effects are mediated by functional connectivity between the hippocampus and posterior insula, primary somatosensory/motor cortex, and PAG—brain regions implicated in the processing of sensory-discriminative aspects of pain. The nocebo-induced connectivity is proportional to the individual anxiety increases. Connectivity between the hippocampus and the amygdala was negatively correlated with the pain intensity. Nocebo-induced anxiety heightened nociceptive input through changes in descending pain modulatory areas.

Specific patterns of brain activity associated with nocebo effects have been reported for itchiness (96) and shortness of breath (97). Negative expectation–induced itchiness is associated with functional connectivity changes between the insula and the PAG (96). Shortness of breath induced by a device inserted into the breathing system under nocebo verbal suggestion is associated with activation of the PAG area (97).

**Figure 4:**
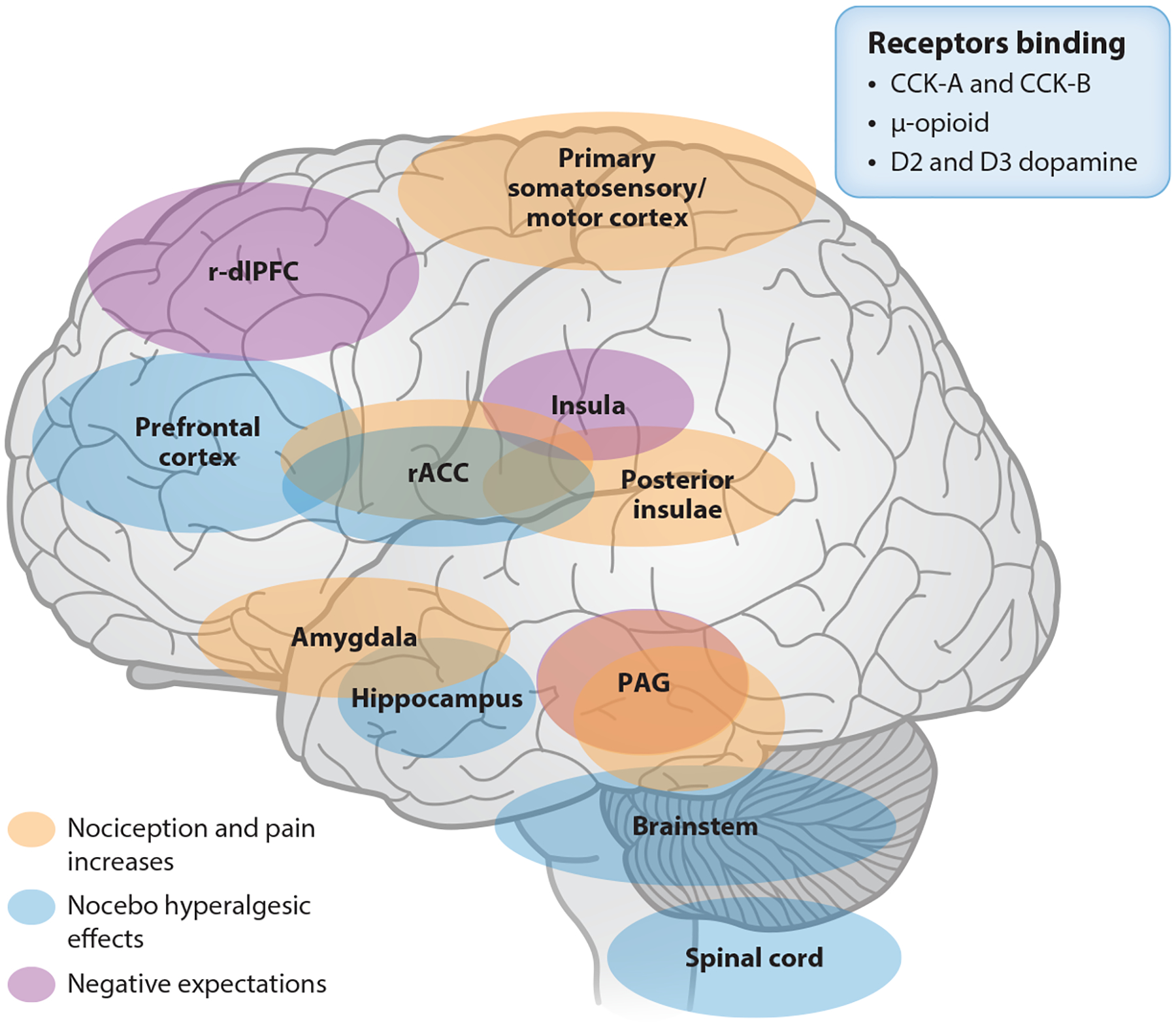
Brain pathways implicated in descending modulation (facilitation) of nociception, negative expectations, and nocebo-related effects. The dorsal horn of the spinal cord receives nociceptive inputs that are regulated by descending control regions such as the rACC, hypothalamus, amygdala, and PAG. The RVM in the brainstem combines these areas to influence nociceptive processing. Prefrontal areas, brainstem, and spinal cord are associated with nocebo-related effects. The hippocampus and regions associated with anticipatory anxiety such as the posterior insula, primary somatosensory/motor cortex, and PAG mediate nocebo effects. The insula and PAG have been also associated with negative expectations. Abbreviations: CCK, cholecystokinin; PAG, periaqueductal gray; rACC, rostral anterior cingulate cortex; r-dlPFC, right dorsolateral prefrontal cortex; RVM, rostroventral medulla.

Overall, these studies have shown that nocebo effects can depend on the order in which treatments are delivered and can induce increased pain, itchiness, and shortness of breath (and other symptoms). Modulatory patterns of brain activity have been associated with these nocebo effects (Figure 4). A recent study used repeated transcranial direct current stimulation (tDCS) and fMRI to determine the modulatory effects of anodal and cathodal tDCS applied at the level of the right dorsolateral prefrontal cortex (98), an area implicated in placebo analgesia (99), on nocebo responses. tDCS decreased nocebo responses induced by an expectancy manipulation model. These results suggest a modulatory role of this region in nocebo pain responses.

### 3.3. Pharmacological Studies of Nocebo Hyperalgesia

Nocebo effects were linked by positron emission tomography with binding of carbon 11 [^11^C]-labeled raclopride and [^11^C]carfentanil to a deactivation of D3/D4 dopamine and opioid release (100). Cholecystokinin (CCK) is a peptide involved in anxiety and panic (101, 102). Benedetti et al. (101, 102) showed that the CCK A/B receptor antagonist proglumide blocks nocebo hyperalgesia. The authors used ischemic arm pain and negative suggestions of pain increases to induce nocebo hyperalgesia in healthy participants. The verbal-induced hyperalgesia was associated with increased adrenocorticotropic hormone (ACTH) and cortisol plasma concentrations. The benzodiazepine diazepam blocked both nocebo hyperalgesia and increased ACTH and cortisol. In contrast, proglumide blocked nocebo hyperalgesia but not increased ACTH and cortisol. Future studies are needed to determine whether CCK is also involved in nocebo effects in chronic pain patients and other patient populations.

Overall, nocebo effects can activate the body’s stress response, leading to the release of stress hormones such as cortisol and adrenaline. These hormones can cause a range of physical symptoms, such as increased heart rate, sweating, and muscle tension. Nocebo effects can also alter the way that the brain perceives pain. Studies have shown that the expectation of pain can activate the same regions of the brain that are involved in processing actual pain, leading to an increased experience of pain. Nocebo effects have been associated with levels of neurotransmitters such as μ-opioids, dopamine, and CCKs. Neurobiological mechanisms of nocebo effects can have a powerful influence on the body and the brain and can lead to a range of negative symptoms and outcomes.

## 4. TRANSLATIONAL RESEARCH ON NOCEBO EFFECTS

Translational research is needed to bridge the gap between basic research and clinical practice. Clinical studies of nocebo effects can help us understand the relevance of the nocebo phenomenon. Ethical considerations are needed to advance current and future endeavors.

### 4.1. Clinical Studies of Nocebo Effects

Clinical studies have shown the effects of negative expectations and nocebo responses on medical outcomes and perceptions of symptoms. A pioneering study by Daniels & Sallie (103) showed that expectations are linked to side effects of lumbar puncture. Of the 15 patients receiving lumbar puncture who were told to expect a headache afterwards, seven experienced headaches. Of the 13 patients who were not warned, none experienced headaches (103).

Other studies were conducted to test whether information delivered prior to treatment regarding side effects and patients’ (clinicians’) expectations can induce paradoxical reactions to treatments, the occurrence of new side effects, and the worsening of medical symptoms. For example, when asthmatic patients received a bronchoconstrictor but were told that the treatment was a bronchodilator, bronchodilation (widening of the bronchi) was observed. Similarly, a bronchodilator resulted in bronchoconstriction when the treatment was presented as a bronchoconstrictor (104). Another paradoxical response to treatment was described with muscle stimulants. When participants were told that a muscle stimulant (a treatment increasing muscle tone) was administered, they experienced muscle tension even though a muscle relaxant (a treatment decreasing muscle tone) was administered (105).

Akin to the ability of informed consents to trigger side effects in those who receive placebos in randomized clinical trials (as described in Section 2), patients can perceive worsening of symptoms by merely being informed about the profile of a treatment’s side effects. Parkinson’s disease patients implanted with deep brain stimulation to the subthalamic nucleus showed improvement of bradykinesia (i.e., slow movements) when they were told that the stimulation was on, when in fact the stimulation was turned off (106). In contrast, when the stimulator was turned on and parkinsonian patients were told that it was off, the misleading information caused worsening of bradykinesia and decreased movement velocity (107, 108).

Patients with benign prostatic hyperplasia who were treated with finasteride experienced different rates of side effects based on the information they received (109). Half of the patients were told that finasteride can cause decreased libido, erectile dysfunction, and problems with ejaculation although these side effects are uncommon. Information about finasteride’s potential side effects was not provided to the other patients. Those informed about the side effects of finasteride had a 43% rate of side effects, while those who were not told about the side effects had a 15.3% rate of side effects (109).

Women in labor were treated with epidural anesthetics, and the anesthetic was described in two slightly different ways (110, p. 868). Some women were told, “We are going to give you a local anesthetic that will numb the area, and you will be comfortable during the procedure.” Other women were told, “You are going to feel a big bee sting; this is the worst part of the procedure.” Women being warned about the “bee sting” and “worst” part of the procedure felt higher pain associated with the procedure. Women who received an anticipation of a positive response to the anesthetic perceived lower pain during the procedure (110).

**Figure 5:**
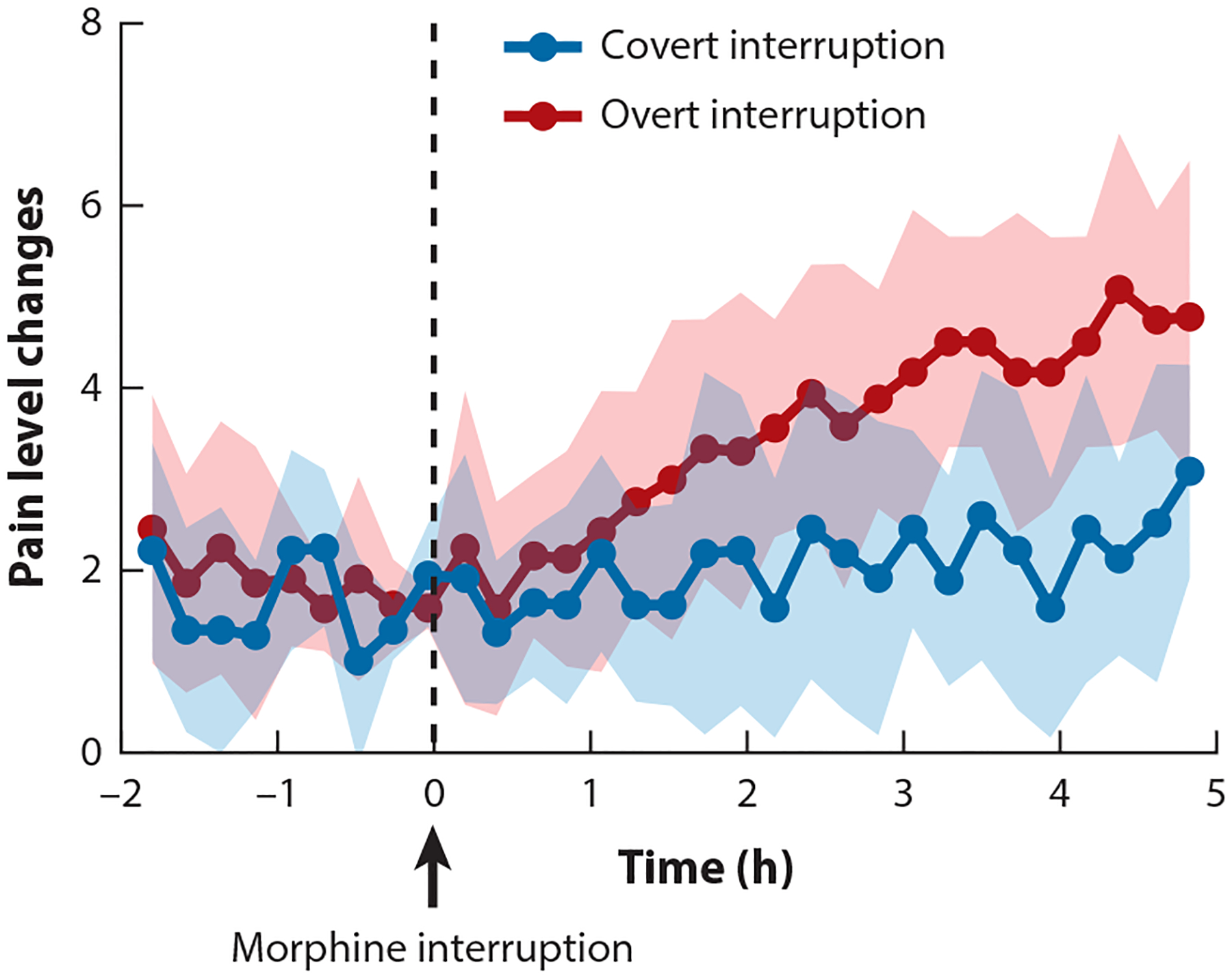
Effects of overt versus covert interruption of morphine administration on clinical postoperative pain. In the overt condition, patients were told that the morphine treatment was stopped, while in the covert condition patients were not told that the treatment was stopped (*upper*). The overt (*red dots*) interruption of the morphine treatment (*dashed line*) increased clinical pain, while the covert (*blue dots*) interruption did not. Numerical rating scores ranged from 0 = no pain to 10 = maximum imaginable pain. Data from Reference 106.

An overt-covert interruption of treatments is a procedure that allows further quantification of the impact of communication and disclosure on medical outcomes in clinical settings (106, 111). Treatments were delivered by means of a computer-controlled infusion pump, and postoperative patients either were or were not told about the time of treatment interruption (Figure 5). Patients overtly informed about the diazepam and morphine interruption experienced an abrupt increase of anxiety and pain, while the covert interruption controlled by computer did not change these symptoms (106).

In early stages of life, nocebo effects can be driven by a history of maternal cortisol levels (112) and early-life stress (113). Infants of diabetic mothers are exposed to cumulative venipunctures to monitor the levels of blood glucose over the first 24–36 h. When different venipunctures for newborn screening are conducted, infants of diabetic mothers showed more pain-related behaviors during the cleaning phase prior to injection than did the normal counterparts. It is likely that anticipatory pain behaviors became a stimulus for nocebo responses (113).

Overall, these clinical observations suggest that nocebo effects can significantly influence treatment responses, procedural interventions, and occurrence of side effects (6, 81). These studies also indicate that communication between patients and health-care providers can inadvertently cause unwanted effects (81, 114) (see the sidebar titled Nocebo Impact on Clinical Treatment and Practice). More generally, interpersonal patient-clinician interactions and environmental aspects of the clinical encounter can promote both positive (115) and negative clinical outcomes, as described above.

### 4.2. Nocebo Effects and COVID-19

Adverse events after placebo treatments are common in randomized clinical trials. This aspect was relevant during the vaccine trials (116, 117) for coronavirus disease 2019 (COVID-19) and may have contributed to vaccination hesitancy (33). Moreover, people’s perception of symptoms (51–53), including long COVID symptoms, and their management (54) can be worsened by negative expectations and beliefs.

The COVID-19 pandemic has been relevant for nocebo research because adverse events after placebo treatments have been observed in COVID-19 vaccine trials. For example, in the Pfizer-BioNTech vaccine trials that included 22,578 placebo and 22,802 vaccine recipients, adverse events such as headache (19.3%) and fatigue (16.7%) emerged in the placebo group following the first placebo vaccine dose (116), suggesting nonpharmacological causes. This is important because these adverse events may have contributed to vaccination hesitancy and people’s perception of symptoms.

Geers et al. (118) conducted a study that showed that prevaccine side effect expectations, worry about COVID-19, and depressive symptoms were the strongest predictors of side effects beyond baseline symptomology, age, vaccine type, and prior COVID-19 infection. Secondary data analyses were conducted for intentions to get a booster vaccine. Booster intentions were related to trust in vaccine development, positive attitudes, concerns about the pandemic, low concern over vaccine side effects, and political (Democratic) affiliation (119). These findings suggest that adequate use of these channels can minimize expectations of side effects for publicly discussed vaccinations and medications for COVID-19 and other diseases (120). The COVID-19 pandemic has also changed the perception of chronic symptoms during the lockdown period, with a shift in attention from chronic pain to anxiety and depression, pointing out the need for mental health care and more comprehensive illness and symptom management (121). This is in line with what has been observed in long COVID, the post-COVID sequelae of symptoms, including fatigue, brain fog, and chronic pain, among others, for which educational and rehabilitation programs are needed to help patients achieve lifestyle changes that can decrease nocebo effects and help recovery processes for long COVID symptoms (122).

### 4.3. Ethical Considerations

Nocebo effects can occur in clinical practice and are related to disclosures of possible side effects from prescribed treatments. Informing patients that prescribed treatments may cause headache can yield this side effect, independent of the treatment’s pharmacological effects. These adverse nocebo effects pose a dilemma for clinicians: The information disclosures can induce adverse side effects, but clinicians have the obligation to convey truthful information to patients so that they can make informed decisions about their treatments in accordance with the principle of autonomy (123).

Can both laws and medical ethics endorse the narrowly circumscribed therapeutic privilege to withhold information? Miller & Colloca (124) described this potential approach in terms of authorized concealment, which could be offered to individuals who choose not to be informed about the side effects profile of a specific treatment (6, 124). In such circumstances, patients could be informed about the nocebo effect and be offered the option to consent not to receive information about the profile of side effects. However, authorized concealment should be limited to mild, transient, and nonsevere side effects. Potential serious, long-lasting, and severe side effects must be disclosed, independent of patients’ values and preferences. Ethically speaking, the consent process can capitalize on optimal clinician-patient communication and framing effects as ways to reduce unintended nocebo effects (4). Clinicians should pay careful attention to the ways information about side effects is disclosed to patients. The likelihood of side effects can be presented using qualitative and/or quantitative modalities to help shape the perception of side effects. For example, information about side effects can focus on the minority of patients who experience particular side effects or on the majority of patients who do not experience such side effects. Tversky & Kahneman (125) showed that people are more likely to undergo surgery if the surgeon tells them the chance of success is 90% versus telling them that the chance of failure is 10%. Different words and terms shape the impact and perception of side effects (126). Thus, truthful information about side effects should be delivered with an acceptable balance between risk-benefit profiles and the patient’s decisions.

Another approach that has been suggested is personalizing the consent process to the patient’s needs to help those who are likely to show nocebo effects (127). This approach is promising, and data-driven research can further inform about this potential way of personalizing medicine. Recently, Clemens et al. (128) tested the desire of being informed about potential side effects in two cross-sectional, between-subject scenario experiments in 999 participants with chronic illnesses (128). When participants learned about a back pain treatment and its profile of side effect severity, frequency, and duration, they reported a high desire for being informed about the side effects. However, the desire to be informed about side effects was reduced when participants were informed that the side effects were less severe and less frequent.

## 5. CONCLUDING REMARKS AND FUTURE DIRECTIONS

Mechanistic research should further elucidate factors that cause the nocebo effect under different conditions, its phenotypes, and neural bases to improve treatment outcomes (114). Nocebo responsivity depends on multiple factors, including gene variations (and potentially expression) (129), learning processes, and personality and psychological factors (9). Additionally, contexts and negative expectations are dynamically updated, making it challenging to develop methods to identify those who are prone to or protected from nocebo effects.

Translational research bridges the gap between basic research and clinical practice. Interventions that target nocebo-related factors, such as patient education, reassurance, and attentional training, have shown promise in reducing nocebo effects in clinical settings (119). However, their effectiveness and applicability in real-world contexts require further investigation. Translational research can also facilitate the development of personalized interventions that take into account individual differences in nocebo responsivity. Ultimately, a better understanding of the mechanisms underlying nocebo effects can lead to improved treatment outcomes and better patient care. There are several recommendations for future studies on nocebo effects. Firstly, studies should explore the impact of nocebo effects throughout a person’s life span, including children and older adults. Additionally, animal studies are necessary to better understand the molecular bases of these effects. Lastly, future studies should focus on identifying clinical phenotypes and other individual factors related to nocebo effects to improve study designs. By addressing these areas of research, we can gain a more comprehensive understanding of the nocebo effect and its impact on human health.

## SUMMARY POINTS

1. Nocebo responses are negative effects observed in patients who receive a placebo in randomized clinical trials and can paradoxically worsen symptoms and cause negative responses to medical treatments.
2. A significant proportion of patients (around one-quarter) who receive a placebo in clinical trials stop taking it due to side effects caused by nocebo responses.
3. Nocebo effects can be induced by factors such as treatment labeling and marketing, which can affect clinical outcomes in randomized clinical trials.
4. Nocebo effects are caused by negative information, prior negative experiences, social influences, and other mechanisms that exacerbate signs and symptoms.
5. Neuroimaging studies suggest that nocebo effects are associated with the activation of descending modulatory systems involving areas such as prefrontal areas, brainstem, and spinal cord.
6. It is important to consider ethical issues related to nocebo responses/effects and take steps to minimize them in clinical encounters and communication with patients (i.e., adequate informed consents).
7. Educational and patient-oriented programs can help frame adverse events in a way that minimizes the negative effects of leaflets and media coverage on symptom perceptions.

## NOCEBO IMPACT ON CLINICAL TREATMENT AND PRACTICE

Nocebo responses have negative effects on treatment adherence and cause adverse side effects and dropouts in clinical trials. Clinical observations suggest that nocebo effects can significantly influence treatment responses, therapeutic decisions, and occurrence of side effects. Communication between patient and health providers can inadvertently cause unwanted nocebo effects.

## Abbreviations

Side effects: expected negative reactions to medical treatment
Nocebo: the term nocebo derives from the Latin verb *nocere*, which means “to harm”
Nocebo reaction: negative counterpart of the positive placebo reaction or response
Nocebo responses: adverse responses to placebo interventions in placebo-controlled trials
Nocebo effects: neurobiological phenomena associated with actual or perceived harm
Negative expectancies: anticipation that future events will be negative
Mass psychogenic modeling: spread of illness through a population in the absence of a physical cause, poison, or contagion agent
Treatment discontinuation: interruption of a medication treatment, which is initiated by either the patient or the clinician (e.g., deprescribing)
Adverse events: unexpected and more severe negative reactions to medical treatment
Biosimilars: generic products (e.g., drugs) typically manufactured by different companies that can be sold once the original patent of the original product expires
Infliximab: monoclonal antibody that enhances and optimizes the immune system
Hyperalgesia: increased sensitivity to pain sensations
Overt-covert procedure: also called open-hidden, refers to the administration (or interruption) of medical treatments with or without a patient’s knowledge
Framing effects: the presentation of an option as a loss or a gain
